# Evaluation of sodium lauryl sulfate for the development of cow-side mastitis screening test

**DOI:** 10.14202/vetworld.2021.2290-2295

**Published:** 2021-08-31

**Authors:** Nobonita Sarker Tanni, Md. Shafiul Islam, Mojahidul Kabir, Mst. Sonia Parvin, Md. Amimul Ehsan, Md. Taohidul Islam

**Affiliations:** 1 Population Medicine and AMR Laboratory, Department of Medicine, Faculty of Veterinary Science, Bangladesh Agricultural University, Mymensingh-2202, Bangladesh; 2 Poultry and Livestock Program (Ultra Poor Graduation Program), BRAC, Barishal, Bangladesh; 3 Department of Livestock Services, Farmgate, Dhaka, Bangladesh

**Keywords:** bovine subclinical mastitis, dairy cows, screening test, sensitivity, sodium lauryl sulfate, specificity

## Abstract

**Background and Aim:**

Subclinical mastitis (SCM) is an economically important disease for dairy cattle worldwide; therefore, regular screening is imperative to detect SCM at an early stage so as to control it. The study was conducted to compare the test characteristics of sodium lauryl sulfate (SLS) as a test reagent to detect SCM in dairy cows.

**Materials and Methods:**

First, 106 milk samples of dairy cows were subjected to available indirect screening tests (white side test [WST], surf field mastitis test, Leucocytest, and Immucell) considering somatic cell count (SCC) as gold standard test. Then 273 milk samples were allowed to react with different concentrations of SLS with or without sodium hydroxide (NaOH) and indicators (bromothymol blue [BTB] and bromocresol purple [BCP]).

**Results:**

SLS with or without NaOH yielded best reaction with the milk samples similar to Leucocytest. It was observed that the reaction of milk samples with SLS added with indicators (BTB and BCP) was easier to visualize than without indicators. SLS 3%+NaOH 1.5% with BTB and SLS 2% with BCP had high sensitivity, specificity, and substantial agreement with SCC. The area under the receiver operating characteristics curve of SLS 2% with BCP and SLS 3%+NaOH 1.5% with BTB was 0.917 and 0.875, respectively.

**Conclusion:**

It may be concluded that SLS 3%+NaOH 1.5% with BTB and SLS 2% with BCP may be the potential reagents for the development of an effective cow-side test to detect SCM, as the main ingredient SLS is considerably cheap and readily available in developing countries.

## Introduction

Mastitis is one of the most prevalent and costly diseases of dairy cows [1]. It occurs in two forms, clinical and subclinical mastitis (SCM) [1]. SCM is the foremost production disease of dairy cows, which can directly or indirectly affect the economy of the farmers globally, including in developing countries [2-4]. Earlier reports indicate that the cost of SCM is often greater than that of clinical mastitis [5,6]. SCM is the inflammation of the mammary gland that does not create any visible changes in the milk or of the udder but the quality and quantity of the milk is highly altered [1]. Both clinical and subclinical forms of mastitis have been considered as the major constraints for the development of the dairy industry in Bangladesh [7]. SCM in dairy cows is important because it (a) is 15-40 times more prevalent than the clinical form, (b) usually precedes the clinical form, (c) is of long duration, (d) is often undetected, (e) reduces milk production, (f) adversely affects milk quality, and (g) serves as reservoirs for the spread of the infection to other cows in the herd [8,9]. SCM causes 1.2-33% milk production losses in affected quarters; however, this loss per farm ranges from 1.3% to 13.5% [10,11]. The reduction in milk production attributed to SCM accounts for 32-70% of the total losses [5,12]. Like other countries, SCM is also a major problem for dairy cows in Bangladesh [13,14]. The prevalence of bovine SCM in Bangladesh is 20.2% [13]. The annual economic losses that occur due to reduced milk production caused by SCM in Bangladesh have been estimated to be Taka 122.6 million (US $ 2.11 million) [7].

Early detection of SCM is the key component of a mastitis control program [15]. SCM is not a problem of individual cow rather a problem at the dairy herd level [6]. Therefore, it is essential to test the dairy herd of cows periodically for the screening of SCM [14]. According to the International Dairy Federation recommendations, microbiological status of the quarters and the somatic cell count (SCC) are the most common tests to detect the changes in the milk due to inflammatory process [16]. Although bacteriological culture of milk samples is considered as the gold standard method to identify intramammary infection (IMI) it does not provide a measure of the degree of inflammation associated with the infection [17]. Since SCM implies inflammation within the udder, but not necessarily infection, the inflammatory reaction due to SCM can be identified by an elevated SCC, a direct screening test [14]. Besides, the SCC is considered as a useful predictor of IMI and an important component of milk to assess the milk quality, hygiene of the herds, and mastitis control program [18-21]. SCC using an automatic counting machine is considered a more accurate direct screening test [22,23]. However, it is expensive and not feasible at the field level to be used by the farmers in the context of Bangladesh. There are some indirect cow-side tests to detect SCM at the field level, such as California mastitis test (CMT), white side test (WST), and surf field mastitis test (SFMT) [16,24,25]. Among all the indirect screening tests, CMT has been considered as the most sensitive and specific screening test [26,27]. The positive reaction of CMT depends on the concentration of somatic cells in the milk [28]. However, in the context of Bangladesh, it is not available whenever needed and is somewhat expensive. The reagent (Sodium alkyl aryl sulfonate) used in the CMT as the leading component is also not readily available in Bangladesh. The ingredients of other tests such as WST and SFMT are available and cheap. However, their sensitivity (*Se*) and specificity (*Sp*) are lower compared to CMT [25,29]. The main component used in CMT (Sodium alkyl aryl sulfonate) is an anionic surfactant which decreases surface tension, changes the structure and conductivity of cell membrane and nucleus, interferes osmotic balance, block oxidization and stimulate proteolytic enzymes, and increase milk viscosity [30]. Like sodium alkyl aryl sulfonate, sodium lauryl sulfate (SLS) may be another potential anionic surfactant with similar functions of sodium alkyl aryl sulfonate to detect SCM [31], which is easily available and cheap.

The study was conducted to compare the test characteristics of SLS as a test reagent to detect SCM in dairy cows.

## Materials and Methods

### Ethical approval and informed consent

Ethical approval was not required for this study because animal experimentation was not done. During milk sample collection, none of the cows were harmed. However, informed written consent was obtained from the owner of the cows to collect milk samples and data. Furthermore, the study protocol was approved by the board of studies of the department, and the Committee for Advanced Studies and Research of the university.

### Study period and location

The study was conducted from February to May 2018. Study cows were selected from Mymensingh Sadar Upazila, and the laboratory works were conducted in the Population Medicine and AMR Laboratory of the Department of Medicine, Bangladesh Agricultural University, Mymensingh.

### Collection and transportation of milk samples

Three hundred and seventy nine milk samples were collected on several occasions from apparently healthy crossbred dairy cows (Friesian × Indigenous and Sahiwal × Indigenous) from smallholder dairy farms of Mymensingh Sadar Upazila. The farms mainly practiced zero grazing. The cows were provided with green grasses such as Napier, roadside grass, and seasonal maize. However, cows were given free access to paddy straw as roughage. Besides, most of the cows were offered varying amounts of concentrates such as rice bran, wheat bran, and occasionally chickpea bran. Most of the farms had the brick floor. The practice of manure removal was on a daily basis. Hand milking was practiced in all the farms. Pre- or post-milking teat dipping was not practiced in any of the farms. Milk samples from cows at 1^st^ week of lactation were excluded due to the presence of physiologically high concentration of somatic cells in milk during this period, which may affect the results of indirect screening tests. Grossly, dirty teats and udders were thoroughly washed, dried, and soaked with 70% ethanol immediately before collection of samples. Three or four streams of milk were discarded to minimize the chance of contamination. Approximately 15 mL of milk was collected from each quarter in a sterilized falcon tube and kept in a cooler box. Then, the samples were transported to the laboratory within 2-3 h, and screening tests and SCC were performed on the same day.

### Study description

Initially, 106 milk samples were tested with four existing indirect screening tests (WST, SFMT, Leucocytest, and Immucell) and gold standard test (SCC). Among them, the Leucocytest with high *Se* was used in the next step. Later, 273 samples were tested with anionic surfactant (SLS) of different concentrations with or without indicators and Lleucocytest. In both steps, SCC was used as gold standard test. All the samples were tested by the first author, who is a skilled registered veterinarian.

### Indirect screening tests for SCM

#### Leucocytest

The test was conducted as per the manufacturer’s instruction (Synbiotics Corporation-2, rue Alexander Fleming-69007 Lyon, France). Briefly, 2.0 mL of quarter milk sample was mixed with an equal volume of reagent in a paddle. The mixing was accomplished by the gentle circular motion of the paddle in a horizontal plane for few seconds, and the changes in milk fluidity and viscosity were observed. The results were interpreted as 0 (negative), 1+ (weak positive), 2+ (distinct positive), and 3+ (strong positive).

#### Immucell (Coburn Company, USA)

To make working solution, CMT concentrate was added to the lower line marked on a dispensing bottle, and then water was added to fill the level line marked at top of the dispensing bottle. Milk samples were taken into four cups of a paddle, and then the paddle was tilted until milk was halfway between the inner and outer circles. CMT working solution was added into each cup slowly. In horizontal position, the paddle was rotated. The results were interpreted as negative (-), trace (T), weak positive (1), distinct positive (2), and strong positive (3).

#### WST

Fifty microliters (five drops) of milk were placed on a glass slide with a blue background. Subsequently, 20 mL (two drops) of 4% sodium hydroxide (NaOH) solution was added to the milk sample and the mixture was stirred rapidly with a toothpick for 20-25 s. The results were interpreted as strong (+++), distinct (++), weak (+), trace (±), and negative (-) based on the formation of coagulation.

#### SFMT

A shallow paddle having four cups was used and rinsed after each use. About 2 mL of milk sample and equal volume of 3% SFMT reagent (Surf Excel^®^ Unilever, Bangladesh) was mixed in the cup of a paddle. Mixing was accomplished by gentle circular motion of the paddle in horizontal position for few seconds. The formation of gel indicated that the sample was positive for SCM. The peak of reaction was obtained within 30 s. The results were interpreted based on the gel formation as negative (-), trace (±), weak reaction (+), moderate reaction (++), and strong reaction (+++). For all the indirect screening tests, milk samples giving weak to strong reactions were considered positive for SCM.

### SCC

Fifty microliters of milk sample and an equal volume of reagent C (1:1 dilution) were taken in an Eppendorf tube using micropipette and mixed using a vortex mixture. Then, SCC-Cassette was loaded with the diluted milk sample by immersing the tip of the cassette into the solution and pressing the piston. The SCC-Cassette was placed in the instrument (NucleoCounter^®^ SCC-100, Chemometec, Denmark) and pressed the “Run” key. After 30 s, the SCC was presented on the instrument display. A milk sample was considered positive when the SCC value was ≥ 100 × 10³ cells/mL [32].

### Anionic surfactant as test solution to detect SCM in dairy cows

SLS was selected as a test reagent for the diagnosis of SCM. Different concentrations of SLS were used with or without NaOH and indicators (bromothymol blue [BTB] and bromocresol purple [BCP]).

#### Test procedure

An equal volume (1 mL) of SLS solutions and milk sample was mixed on a shallow paddle having four cups, and the mixing was properly done by the gentle circular motion of the paddle for few seconds. The reaction developed almost immediately with milk containing a high concentration of somatic cells. The peak of reaction was obtained within 10 s and any change in the milk, such as viscosity, consistency was observed as mentioned below.

**Table:** 

Reaction characteristics	Score	Infection
Consistency and viscosity are normal.	Negative (0)	Absent
Very slight thread formation and/or precipitation disappearing after stirring	Trace (±)	Slight infection risk
Distinct precipitation and threads formation but no gel formation	Weak positive (+)	Subclinical mastitis
Mixture become more viscous and formation of gel	Distinct positive (++)	Subclinical mastitis
Distinct gel formation, which is thick and has tendency to adhere to the bottom of the cup of the paddle	Strong positive (+++)	Subclinical mastitis near the clinical stage

### Statistical analysis

SCC was considered as the gold standard test for the determination of epidemiological test characteristics of different indirect screening tests as well as test anionic surfactant - SLS. Five quality parameters: *Se*, *Sp*, positive predictive value (PPV), negative predictive value (NPV), and kappa-value were calculated. The results were weighted on the basis of *kappa* value, which ranges from 1 to –1, where a value of –1 indicates complete disagreement between tests, 0 indicates agreement by chance only, 0.01-0.20 indicates slight agreement, 0.21-0.40 indicates a fair amount of agreement, 0.41-0.60 indicates moderate agreement, 0.61-0.80 indicates substantial agreement, and 0.81-1 indicates almost perfect agreement [33]. The SPSS v.20 was used for all the analyses.

### Receiver operating characteristic (ROC) curve

A graph of *Se* against 1-*Sp*, that is, a ROC curve was generated and the performance of a diagnostic test was quantified by calculating the area under the ROC curve (AUROC). The ideal test would have an AUROC of 1.

## Results

### Comparison of existing indirect screening tests with SCC

Four popularly used indirect screening tests (Leucocytest, WST, SFMT, and Immucell) along with SCC as gold standard test were employed on 106 milk samples of dairy cows to diagnose SCM and to compare the test characteristics. Leucocytest had comparatively higher *Se* (64.9%) than that of WST (45.9%), Immucell (56.8%), and SFMT (63.9%) (Table-1). Kappa-value indicated substantial agreement of Leucocytest with SCC. Therefore, Leucocytest was selected for further comparison with test anionic surfactant SLS.

**Table-1:** Sensitivity, specificity, and agreement of four indirect screening tests with somatic cell count for the diagnosis of subclinical mastitis (n=106).

Screening tests	*Se* %	*Sp* %	PPV %	NPV %	Kappa-value
WST	45.9	98.6	97.4	77.5	0.505
SFMT	63.9	85.1	69.7	81.4	0.499
Leukocytes	64.9	95.7	92.5	84.1	0.646
ImmuCell	56.8	80.8	80.4	81.0	0.455

n=Number of milk samples, WST=White side test, SFMT=Surf field mastitis test, Se=Sensitivity, Sp=Specificity, PPV=Positive predictive value, NPV=Negative predictive value, Kappa-value=Measurement of agreement

### Evaluation of SLS at different concentrations

**Figure-1:**
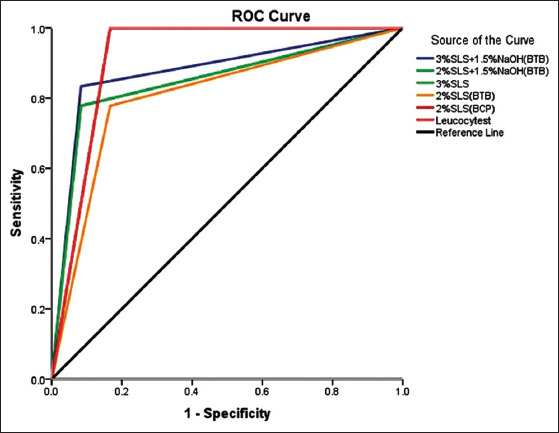
Receiver operating characteristic curve for different tests performance.

SLS with or without NaOH yielded the best reaction with the milk samples similar to Leucocytest. It was observed that the reaction of milk samples with SLS added with indicators (BTB and BCP) was easier to visualize than without indicators. *Se*, *Sp* and kappa-value of SLS 3%+NaOH 1.5% with BTB and SLS 2% with BCP were higher than other concentrations of SLS (Table-2). The *Se* and *Sp* of both Leucocytest and 2% SLS with BCP was 1, and the area under the curve (AUROC) for both the tests was 0.917, which was higher than AUROC of other concentrations of SLS (Figure-1). The AUROC of 3% SLS+1.5% NaOH with BTB was also high (0.875). As SLS 3%+NaOH 1.5% with BTB and SLS 2% with BCP gave the best reactions in terms of *Se*, *Sp* and diagnostic agreement they can be potential reagents to be used as indirect screening tests to diagnose SCM in dairy cows. The test results are further summarized in Table-3.

**Table-2:** Sensitivity, specificity, and agreement of sodium lauryl sulfate with and without indicators with somatic cell count for the diagnosis of subclinical mastitis (n=273).

Screening tests	*Se* %	*Sp* %	PPV %	NPV %	kappa-value
SLS 3%	77.8	91.7	93.3	73.3	0.667
SLS 3% with BTB	61.1	100	100	63.1	0.557
SLS 3%+NaOH 1.5%	83.3	91.7	93.7	78.6	0.730
SLS 3%+NaOH 1.5% with BTB	83.3	91.7	93.7	78.6	0.730
SLS 2%	66.7	100	100	66.7	0.615
SLS 2% with BTB	77.8	83.3	87.5	71.4	0.595
SLS 2% with BCP	100	83.3	90	100	0.857
SLS 2%+NaOH 1.5%	72.2	100	100	70.6	0.675
SLS 2%+NaOH 1.5% with BTB	77.8	91.7	93.3	73.3	0.667
SLS 2%+NaOH 1.5% with BCP	72.2	83.3	86.7	66.7	0.533
Leukocytes	100	83.3	90	100	0.857

n=Number of milk samples, SLS=Sodium lauryl sulphate, BTB=Bromothymol blue, NaOH=Sodium hydroxide, BCP=Bromocresol purple, *Se*=Sensitivity, *Sp*=Specificity, PPV=Positive predictive value, NPV=Negative predictive value, Kappa-value=Measurement of agreement

**Table-3:** Sensitivity, specificity, and agreement of sodium lauryl sulfate with indicators with somatic cell count for the diagnosis of subclinical mastitis.

Screening test	SCC				

	*Se* %	*Sp* %	PPV %	NPV %	kappa-value
SLS 3%+NaOH 1.5% with BTB	83.3	91.7	93.7	78.6	0.730
SLS 2% with BCP	100	83.3	90	100	0.857
Leukocytes	100	83.3	90	100	0.857

SLS=Sodium lauryl sulfate, BTB=Bromothymol blue, NaOH=Sodium hydroxide, BCP=Bromo cresol purple, *Se*=Sensitivity, *Sp*=Specificity, PPV=Positive predictive value, NPV=Negative predictive value, Kappa-value=Measurement of agreement

## Discussion

SCM is not a problem of individual cows rather a problem at the dairy herd level; therefore, it is essential to test the dairy cows of the herd periodically for the screening of SCM [6]. *Se* and *Sp* are intrinsic values of a test and are useful for comparing one diagnostic test with another [34]. However, they cannot be used to determine the probability that a given test result reflects the true disease status of the animal [35]. *Se* and *Sp* can be calculated only if there is an objective, independent means of determining whether the disease is truly present [34].

Here, an anionic surfactant, SLS was evaluated based on the *Se* and *Sp* taking SCC as the gold standard for the detection of SCM in dairy cows. SLS with or without NaOH at different concentrations gave better reaction with the milk samples similar to existing indirect screening test, Leucocytest. SLS decreases surface tension, change the structure and conductivity of cell membrane and nucleus, interfere osmotic balance, block oxidization and stimulate proteolytic enzymes, and increases milk viscosity. NaOH degrades somatic cells; as a result, the DNA of the cells reacts with the solution and forms gel, which indicates the presence of SCM. Earlier, SLS was evaluated to be used as CMT substitute; however, the test properties and interpretation of results were not clearly explained [16,29]. The high *Se* of a test indicates the proportion of total infected quarters that are truly infected. The *Se* of SLS solutions was very much close and similar to the Leucocytest. It was also observed that the *Sp* of the SLS solutions was also high, which means that it is able to detect uninfected samples which are not truly infected. These highly satisfactory intrinsic values of the SLS solutions increased the acceptability of this solution to be used as a test solution to detect SCM in dairy cows.

In addition, PPV and NPV were also high, which indicates that SLS identified milk samples from truly affected quarters as positive and from truly not-affected quarters as negative. Further evaluation was done by adding indicators to SLS to observe the reaction of milk. Indicator is a halochromic chemical compound added in small amounts to a solution so that the reaction of the solution can be determined visually [36,37]. Therefore, SLS with or without NaOH were subjected to further evaluation with or without indicators (BTB and BCP). It was observed that the reaction of milk samples with SLS added with indicators (BTB and BCP) was easier to visualize than without indicators. *Se*, *Sp* and kappa-value of SLS 3%+NaOH 1.5% with BTB and SLS 2% with BCP were higher than other concentrations of SLS. Among the available screening tests, the *Se* was very low in case of WST and SFMT. Muhammad *et al*. [24], Sarker [38], and Badiuzzaman *et al*. [25] also reported the lower *Se* of WST and SFMT. Although Leucocytest had high *Se* and *Sp* it is somewhat costly as well as not readily available whenever needed in our country. On the other hand, WST and SFMT are not appreciated due to its low *Se*.

## Conclusion

SLS 3%+NaOH 1.5% with BTB and SLS 2% with BCP may be the potential reagents to be used as test solutions to detect SCM in dairy cows as SLS is available and relatively cheap in our country. However, field evaluation of these reagents is needed to validate their characteristics as a screening test to detect SCM in dairy cows.

## Authors’ Contributions

NST and MTI: Conceptualization. NST, MSI, and MK: Sample collection, data collection, and data curation. NST, MSI, MK, and MSP: Formal analysis. MTI: Funding acquisition. NST: Investigation. NST: Methodology. MTI and MAE: Project administration. NST, MSP, and MTI: Resources. MTI and MAE: Supervision. NST, MSP, and MTI: Validation and visualization. NST: Writing – original draft. NST, MSI, MK, MSP, MAE, and MTI: Writing – review and editing. All authors read and approved the final manuscript.
